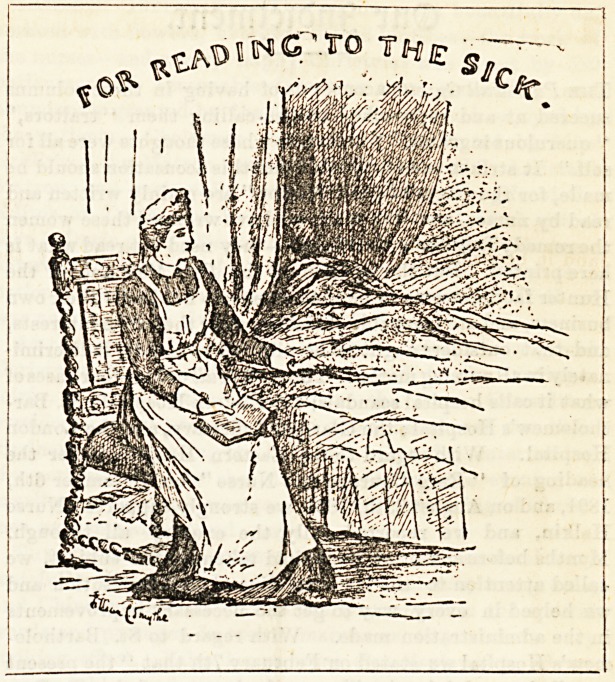# The Hospital Nursing Supplement

**Published:** 1892-01-09

**Authors:** 


### "The Hospital" Nursing Mirror.

BEING THE EXTRA NURSING SUPPLEMENT OF "THE HOSPITAL" NEWSPAPER.

Contributions for this Supplement should be addressed to the Editor, THE HOSPITAL, 140, Strand, London, W.C., and should have the word "Nursing" plainly written in left-hand top corner of the envelope.

### En Passant.

ST. ANDREW'S AMBULANCE ASSOCIATION.—This is practically the St. John's Association of Scotland; it provides waggons, or, as we should call them, ambulances, which last year conveyed 2,346 cases, and it gives lectures which last year trained 4,180 pupils. There are twenty-seven centres in connection with the Association, which occupies a firm position in Glasgow and Edinburgh, and is financially a great success. Dr. George T. Beatson, of Glasgow, has just published a most excellent ambulance "handbook" for the use of pupils, which we hope to review fully next week. Meanwhile, we would note that the Scotch Association seems to work on quieter and more economical lines than the St. John's Association, and yet to be equally successful.

SHORT ITEMS. — The nurse-dolls are going to be exhibited at Bath in February.—From 50 to 60 cases are being refused daily at the Nurses' Co-operation, sure sign of the great increase of sickness just now.—Male nurses have started a co-operation to themselves at 20, Westmoreland Street. — Charlotte Elizabeth Norree has patented an improvement in spinal supports, and Florence Amelia Fairthorne an improvement in impervious fabrics for bandages.—The Treasurer of the Welsh branch of the Queen Victoria Nurses complains of the apathy with which the institute is treated in Wales.—Mr. Springfield, whose son had his leg broken on the Great Eastern Railway, said in court "I must really take this opportunity to thank the Matron of the Beccles Hospital. Such a woman it has rarely been my pleasure to meet, and the way in which she treated those patients should be recognised."—Many communications are unavoidably held over this week owing to the pressure on our space.

THE ROYAL NATIONAL PENSION FUND FOR NURSES.—A most successful meeting was held on Saturday afternoon in Dublin Castle at the invitation of the Countess of Zetland. The meeting, which was attended by officials and nurses of most of the larger Irish hospitals, was addressed by Mr. Burdett, who explained the objects of the Fund. The Right Hon. Lord Justice Fitzgibbon, Chairman of the Dublin Nursing Institution, took the chair on the occasion, and in a short introductory address, made fitting allusion to the interest taken by the Countess of Zetland in the Fund, of which she is now a lady patroness, and in all good works. The President of the College of Surgeons, in moving a vote of thanks to Mr. Burdett, remarked that it would be an excellent thing if the wages of Irish nurses, who had so increased in efficiency, could be raised, that they might be in a position to help themselves. The Master of the Rotunda Hospital, in seconding the motion, explained the strange fact that a pension fund had not existed previously by remarking that there were no nurses who had any right to the name to be pensioned. Formerly all the qualifications necessary for a nurse were a certain amount of old age and corpulence. Dr. Grimshaw, Registrar-General, gave his testimony that the fund was a sound financial undertaking and not merely a benevolent institution. The Chairman in closing the proceedings, pointed out how advisable it was for nurses to join at the earliest possible age, when the premium was its lowest. He warmly advocated the affiliation scheme for institutions, as assisting to remove the difficulty so universally felt of adequately pensioning or dismissing old servants. Lastly, he expressed a wish that when next the nurses received their certificates from H.R.H. the Princess of Wales, that Irish nurses would be more largely represented.

NURSING NOTES.—We have much pleasure in giving a hearty welcome to our monthly contributor "Nursing Notes" in a new cover with which it commences the year 1892. It is a most readable little paper, well edited, and excellently conducted. Its advertising columns show that it is succeeding as it deserves to succeed, and we heartily congratulate the enterprising spirits who manage the Workhouse Infirmary Nursing Association and the Midwives' Institute and Trained Nurses' Club, upon the production of this practical journal for nurses.

CHRISTMAS PARCELS.—Miss Monk, of King's College Hospital, writes: "May I ask you to express our deep and grateful thanks to the kind friends who have sent us such nice gifts for our poor sick this Christmastide." The Matron of the Seamen's Hospital writes to thank our readers "for the suitable presents received for the sailors; they are exactly what they will most appreciate." Miss Medill, of St. Mary's, writes: "The kind and seasonable gift of warm clothing will be invaluable to our patients in this terrible weather. The garments are most beautifully made." Other kind letters have been received, but the above are enough to show our readers how their work has been appreciated.

THE CHARGE AGAINST THE TRAINING SCHOOLS. —In the New York *Medical Record* for December 19th, there is a story of a savage onslaught on Nurse Training Schools by Mr. Peck, whose daughter died of phthisis, contracted, he believes, from a patient. Says Mr. Peck: "Do you know that more than half the trained nurses die of consumption? Do you know that from one to five years is quite likely to end your life with some vital disease if you choose this occupation? Do you know that if you pass through the two years of training you are likely to be a physical wreck afterward, and your life a burden to yourself and friends?" No; we reply, we know nothing of the sort, and such reckless statements are true neither of America nor England; they injure the man who makes them, not the schools they are urged against. Of course it is no use for a woman to become a nurse if she is afraid of infection or death; a nurse has to risk her life if she pursues her calling fully; so does a doctor; so does every sailor who goes to sea; so does every married woman. But statistics prove that, as a rule, a nurse reaches an average length of life, and if she is properly careful of her own health she can fairly look forward to acheery old age. Unfortunately nurses are not always careful of their health; they become absorbed in the tremendous work of their wards; they refuse the rest offered them; they grow careless about contagion. But this is not the fault of the schools, but of the enthusiasm and self-sacrifice which rise in a woman's heart when she has found the blessing of ministering to others; many a Matron finds that her hardest duty is to get her Sisters to take enough rest and exercise in the open air. Besides, what rubbish it is to talk of hospitals as though they are prisons; if a nurse does not like, or is not equal to the work she can go, and everybody connected with the institution will be heartily glad to get rid of her. Grumbling nurses and delicate nurses are the greatest mistake in the world; they should always be asked to resign at the earliest opportunity, that they make room for strong, efficient, cheery women, whose very presence will bring health to the patients. Nothing exhausts a patient and retards his recovery so much as to be waited on by a weary, unwilling nurse. We are sorry for those women who have the wish to be nurses but not the health; but there are other ways of ministering to the poor and needy where they can fulfil their worthy aspirations.

### Lectures on Surgical Ward Work and Nursing.

By Alexander Miles, M.D. (Edin.), F.R.C.S.E.

### Lecture XLI.—INSTRUMENTS USED IN URETHROTOMY.

There are certain cases in which stricture of the urethra does not yield to treatment by means of dilatation with bougies, and it becomes necessary, or at least advisable, to cut the strictured part of the canal and so restore its calibre. This

Fig. 1.

Fig. 2.

Fig. 3.

operation is known as urethrotomy. Now the stricture may be attacked from the inside of the urethra, in which case we speak of internal urethrotomy; or the cut may be made through the skin—external urethrotomy.

When the incision is made from the inside a very fine blade is introduced, and with great care is made to cut only the strictured part. To ensure accuracy and safety many instruments have been devised and great ingenuity displayed. Some of these cut the stricture from before backwards, that is, on the way into the bladder, such, for example, as Civiale's. Another is that of Maissoneuve This consists in (1) a fine filiform bougie, which can be passed through a very tight stricture; (2) on to this is screwed a small staff, in a hollow in which runs a stylet bearing a triangular shield. This triangle, or wedge, renders the stricture tense, and from within it is pushed a sharp cutting knife edge, which splits the constricting fibres, and so re-establishes the lumen of the canal. Any fibres which may escape division as the instrument enters may be dealt with as it is withdrawn. This instrument has been modified and improved by Teevan. A third instrument for the same purpose has been devised by Mr. Berkeley Hill.

Another set of instruments are arranged to cut the stricture from within outwards, but obviously very tight constrictions cannot be so dealt with, as such would not admit of the entrance of the staff with its concealed knife, and some of the instruments are so large, that if they can be passed at all it almost argues that no operation is necessary. Of instruments cutting from within outwards may be mentioned Sir Henry Thompson's modification of Civiale's instrument. It has a bulbous point, from which a blade projects (Fig. 1). Dr. P. Heron Watson, of Edinburgh, has also introduced an instrument working in this direction. Otis, of New York, has a complicated instrument, consisting of two blades, which are separable, and so can render the urethra quite tense, and when so the knife is protruted and very readily splits the constricting fibres.

The operation of external urethrotomy is performed by the aid of a guide which is passed into the urethra, and being felt through the skin, is cut down upon. The most frequently used staff is that of Syme (Fig. 3a). The curved part of this instrument is about the size of a No. 2 bougie. About three inches from the point it suddenly expands to the size of a No. 10 or 12, thus making a distinct shoulder on the instrument. The thin part is grooved on the convexity, the groove just reaching on to the shoulder and stopping there. The instrument is passed into the urethra with the patient in the lithotomy position, and the shoulder is caught at the stricture. The surgeon then cuts down upon the shoulder, introduces the point of his knife into the groove, and cuts through the stricture in a direction from within outwards. The staff is withdrawn, and an S-shaped silvered catheter introduced through the perineal wound, and left for three or four days

Fig. 4.

Fig. 5.

for drainage. The further treatment consists in regularly passing bougies at stated intervals to prevent recontraction of the stricture.

Mr. Wheelhouse performs external urethrotomy by cutting down upon a grooved staff, which only reaches to the stricture, and then from the perineal wound he passes a fine probe pointed director through the stricture, and on this divides the tight fibres. Drainage is carried out by a silver catheter passed through the meatus, and the perineal wound is allowed to heal up. Sometimes no instrument can be got through the stricture, and the urethra beyond has to be searched for in the peunaem and opened, an operation of extreme difficulty.

URETHRAL DILATORS.—It is sometimes advisable to rapidly and forcibly stretch a strictured urethra, and there are several methods of doing so. (1) By means of sliding tubes. The instrument used by Wakley may serve as a type of this variety. It consists of a fine "urethral guide," which is first passed through the stricture, and then over this a larger tube is passed, and so on, one tube over another till the urethra is expanded to its normal size. (2) Sir Henry Thompson's dilator (Fig. 2) consists of two blades, which may be separated by a screw at the handle. The dilatation should be very slowly effected, a few seconds elapsing between each two turns of the handle. (3) Holt's urethral dilator acts on the principle of a wedge.

It is a split sound, having a fine steel wire between the two halves of the blade. The instrument is passed into the

FIG. 6.

FIG. 7.

urethra, and when there, one of a graduated set of wedge-shaped tubes is passed over this wire, and so forcibly separates the halves of the sound. It is claimed by the introducer that only the strictured part of the urethra is affected by the proceeding. This method of treating stricture is not free of danger, and in most cases may be replaced with advantage by either gradual dilatation or urethrotomy.

URETHRAL FORCEPS (Fig. 4) have long, narrow blades, and are used to remove impacted calculi or other foreign bodies from the urethra. Various instruments are or have been used to apply substances locally to the mucous membrane of the urethra. For example, solid caustics may be applied by the aid of the apparatus figured in Fig. 5; fluid caustics with another; and ointments by a third, which is fluted all round to receive the ointment.

Of all the urethral syringes in use, perhaps that of Balmano Squire (Fig. 6) is the most convenient. It holds just as much as the urethra does, and is made of india-rubber with a glass nozzle, which has an india-rubber cap acting as a stopper. In the instrument figured (Fig. 7), the fluid passes towards the meatus, and is so calculated to wash out the urethra efficiently.

The illustrations are used by kind permission of Messrs. Arnold and Sons.

### ONE AMONGST US.

It makes a very great difference in our lives if there is one person in particular among our friends whom we care for above all the rest, for whom we forget ourselves entirely when they are present, and in their absence try to do everything we know would please them.

This is true unselfish love which asks for no return, but none the less delightful is it to find out suddenly, that our efforts to please are appreciated, that our poor services are acknowledged, that when we thought ourselves overlooked, the object of our esteem and reverence had noticed all our self-restraint, the denial of our wills, and we are rewarded by an equal return of affection.

No we who love God are exactly in that position towards Him. We have tried to the best of our ability to keep His commandments, and please Him by submitting to His will and our hearts begin to faint, because we imagine He leaves us to bear our sufferings alone. But, dear friends, if we love our Saviour, we will not give up, but take one step further, and by faith see that there is One standing among us Who ever smiles on and approves the efforts of His children. He knows we wish to bear our sufferings with patience, but that our puny strength is unequal to the strain, and so we are discontented and peevish when our will is to be grateful and contented. "The spirit indeed is willing, but the flesh is weak," said our dear Lord of His sleeping disciples, and He makes the same allowance for us now, if our hearts are fixed on Him.

But let us return again to the thought of the earthly friend whom we so highly value, and recall the first time we saw him or her.

We were lying in misery possibly, racked with pain in every limb, when a gentle step drew near—it might have been of doctor or nurse—then the soft voice, the gentle touch, the word of sympathy thrilled through our hearts, we felt sudden peace and rest, the pain was bearable, and we seemed to receive fresh strength to endure.

And shall I tell you who was speaking with that voice, who gave you this unexpected ease and comfort? It was Christ. His was the genele touch, the soft, sweet word of sympathy. He had been watching us, for He is ever standing amongst us, eager to heal our wounds and carry our sorrows.

Nineteen hundred years ago, as at this time, He laid aside His glory, and came as a babe into the world for our redemption, but only a few humble hearts knew Him. For three and thirty years His blessed feet trod this earth, carrying peace and healing to all, yet He was derided and rejected. We will not be blind and refuse Him, now that He still stands among us, working by His Word, by His ministers, by all who tend the sick and suffering. Yes, He is about our bed and about our path, and, if we ask Him, will make all our beds in our sickness.

### Our Indictment.

The *Pall Mall Gazette* accuses us of having in these columns sneered at and reproached nurses calling them "traitors," "querulous ingrates," "creatures whose thoughts were all for self." It strikes us as laughable that this accusation should be made, for the pages of the "Mirror" are mainly written and read by nurses, and if in truth we have wronged these women the remedy is in their own hands—they need not read what is here printed. But the opinion of the *Pall Mall* and of the Hunter faction seems to be that nurses do not know their own business, and are incapable of looking after their own interests, and that outsiders ought to be allowed to meddle indiscriminately in all nursing matters. The *Pall Mall* quotes four cases of what it calls hospital scandals; the Eastern Hospital; St. Bartholomew's Hospital; the Glasgow Infirmary, and the London Hospital. With regard to the Eastern Hospital under the heading of "Rare Courage of a Nurse" on December 6th, 1891, and on August 29th, 1891, we strongly supported Nurse Halkin, and we reported fully the enquiry all through. Months before any other paper had taken up the subject, we called attention to the lax discipline at that institution and we helped in every way to get the necessary improvements in the administration made. With regard to St. Bartholomew's Hospital we stated on February 7th that "the present so-called scandal is the illness of nineteen of the St. Bartholomew nurses. Once more, it is not the nurses who complain; it is outsiders who love to rake up charges against philanthropic institutions. No rôle in life in easier than to look on and find fault; no rôle is more contemptible. The amount of illness amongst the St. Bartholomew nurses is greatly to be regretted. . . . Our sympathy is very strongly with the authorities of St. Bartholomew's in their present trying position; and once more we glory in the fact that the nurses themselves are loyal to their *alma mater*, and that the charges receive no confirmation from those within the walls." We search in vain through the paragraph for the terms of abuse we are accused of having levelled at the nurses. With regard to the Glasgow nurses, our strongest expression of opinion was on October 3rd, when we wrote; "If any of the nurses really wrote the letters which have been appearing in the *Mail*, we are shocked at the disloyal tone and carping spirit they have fostered in their midst; but the letters betray such ignorance of nursing matters that we are inclined to believe them the work of outsiders, in spite of the fact that they are all signed by some such title as 'A Ward Nurse.' Even after the managers have instituted an enquiry and are doing their best to learn the wishes of the nurses, the letters continue to appear in the paper. This shows a lack of courtesy all round, and is most unfair. We appeal to all loyal and lady-like nurses in Glasgow to try and secure redress without lowering themselves further in the eyes of their fellow-workers." When the official report came out, and there was evidence of the overworking of the nurses we wrote: "These are legitimate causes of complaint, and the managers in their report have promised the desired amendments. Obviously we have yet to hear the conclusion of the whole matter. The Committee cannot sit silent under the serious and painful accusation that they have persistently worked some of their nurses 24 hours out of the 36." With regard to the London Hospital, we had the pleasure of being present at the presentation by nurses, both past and present, to the Matron, and have nothing but praise for them, which we have persistently expressed. We know well that the efforts to stir up ill-feeling in their midst have failed completely, save that the nurses, were they not ladies, would show their dislike of the meddling in nursing matters by certain women who have the audacity to boast of their ignorance of those things about which they presume to offer advice. For ten years we have watched with pleasure and interest the steady advance of the London as a training school for nurses, and we always chronicle the excellent appointments won by its workers. We fail to find anywhere in our pages the wholesale abuse of nurses of which we are accused; instead we find both praise and practical help given to the efforts to secure nurses fuller pay, shorter hours, and provision for their old age. We have tried to put before nurses the fact that they must put their patients' interests first, their own interests second; and no good nurse would ever dream of doing otherwise; but we have not neglected the interest of nurses. We absolutely deny the accusations of the *Pall Mall*, and defy that paper to prove its statements. The thing is absurd. One of our leading objects as a paper is to help nurses to the best of our ability, and to help them in those ways which our practical acquaintance with nursing work and with hospital management shows us to be desirable. Those who have never done a day's ward work, started an institution, or struggled with the dangers of superintendence, or the difficulties of the secretarial work of a hospital, may howl in print that these duties are not perfectly performed in this or that hospital. Our reply is that we know it; that perfection exists nowhere; and that talking won't produce it. Again we repeat no rôle in life is so easy, and so contemptible as that of the non-workers of the world who sit at their ease and criticise the labours of those who have borne the heat and the burden of the day.

### Appointments.

[It is requested that successful candidates will send a copy of their applications and testimonials, with date of election, to The Editor, The Lodge, Porchester Square, W.]

The Eye Infirmary, Wolverhampton.—Miss Florence Newton, who was trained at the St. Marylebone Infirmary, Notting Hill, has been appointed Matron.

Cottage Hospital, Trowbridge.—Miss Harriet Perkins has been appointed Nurse-Matron to the Cottage Hospital, Trowbridge. Miss Perkins trained in Birmingham, and has had charge of the Eye Infirmary, Wolverhampton, and of the Cottage Hospital, Ross.

Rotunda Hospital, Dublin.—Miss Lucy Ramsden has been appointed Assistant-Matron to the Rotunda Hospital, Dublin. Miss Ramsden trained at St. Thomas's Hospital, and has been a Sister at Monsall Fever Hospital, Manchester, for the past year and a-half, having had (for the greater part of the time) charge of a division containing 100 beds. We congratulate Miss Ramsden, and believe the Rotunda Hospital will have every reason to rejoice at the appointment.

Her Majesty has approved the following nurses being placed on the roll of "Queen's Nurses" for nursing the sick poor in their own homes. Superintendents: Jessie Buchanan Gray, South London; Dora Carter, East London. Nurses: Florence Kidd, Bolton; Mabel Winifred Cross, Mortlake; Agnes Mary Cecilia Burkett, Bloomsbury; Ellen Frances Wood, Chelsea; Winifred Noble, Haggerston; Hannah Rowley, Alfreton; Edith Helen Yate, Cardiff; Charlotte Reeve, Bloomsbury; Emily Kate Heygate, Bloomsbury; Abigail Wratten, Bermondsey; Lillie Steele, Camberwell; Annie Lucy Gardner, Harlow; Mary E. Martin, Manchester; Annie Barber, Manchester; Jane Glass, Manchester; Mary Trotter, Manchester; Harriet Smith, Hamilton; Ellen Rae, Edinburgh; Jemima Macdonald, Glasgow; Ada Leigh, Glasgow; Helen Reid, Glasgow; Elizabeth Pittendrigh, Glasgow; Agnes Brydon, Glasgow; Jessie Blanche Monckton, Dundee; Ada Annie Donaldson, Edinburgh; Sara Keay, Edinburgh; Marian Singleton Burford, Dundee; Emma Catherine Nicholson, Edinburgh; Adelaide Louisa Eyre, Edinburgh; Annie Stewart Cameron, Edinburgh; Constance Jane Woodrow, Dundee; Mina Thompson, Edinburgh; Florence May Smith, Edinburgh.

### Keeping Christmas.

THE LONDON HOSPITAL.—Christmas at this, the largest of the metropolitan hospitals, which stands out alone as the great general hospital of East London, has been, as usual, widely observed to the great delight and intense enjoyment of the 650 patients in the wards. The wards have been extensively and in some places beautifully decorated, and a general air of contented happiness has prevailed in spite of the severity of the majority of cases brought to this institution. Various entertainments, theatricals, concerts, recitations, and small shows were held in the separate wards on Christmas Day, and on Boxing Day the Christmas dinners were provided for the large nursing staff, which now numbers no less than 257. January 5th was appointed as the day for the Christmas trees held annually in Queen Victoria and Princess Beatrice wards. The wards are so named in honour of the visit of Her Gracious Majesty the Queen to open the Grocers' wing in 1876. The centre of both wards was filled with children's cots, and all children in the hospital under 14 years of age were moved into these cots. Of the 150 children who received presents, no less than 74 were under seven years of age. The two large trees were given by Mr. Robert Barclay, a vice-president, and were crowded with toys. Many were sent by Her Serene Highness the Princess Mary of Teck, a large store came from "Truth," from Mr. Leopold de Rothschild, and many other friends. There was a large company present on the invitation of Miss Lückes, the Matron, who had been entirely entrusted with the management of the festivity. Many visitors afterwards visited the wards and adjourned to tea in the Nursing Home. The immense amount of work done at this great charity may be understood from the fact that over 9,500 in-patients are admitted and treated at the hospital every year, while the number of out-patients raises the persons applying for help to over 2,000 per week. Help is urgently needed as no less than £15,000 is required before the end of this month for the settlement of claims due, and the Committee earnestly appeal for donations and new annual subscriptions which will be most thankfully received.

AT GUY'S HOSPITAL the decorations were very pretty, and the "Guy's Ethiopians" visited each ward in turn and gave entertainments; the nurses also sang and helped to amuse the patients. In Lydia Ward and Charity Ward coloured glass lamps in large numbers, together with a profusion of Chinese and Japanese lanterns, produced very pleasing effects. The children's cots had cotton-wool canopies with robins at the summit, and other snow effects were observable wherever they could be placed to advantage. Generally speaking, the use of cotton-wool was discouraged this year on account of the risks of fire. In Dorcas and some other wards there were large Christmas trees laden with presents. On first waking on Christmas morning each patient in QUEEN CHARLOTTE'S discovered by her bedside a warm suit of clothes for her newly-born baby and a warm wrap for herself. These valuable and useful presents were acquired through the generosity of several kind ladies who had the clothes made up for this special purpose. The morning service was conducted in the pretty little Hospital Chapel, where the nurses and servants attended. At noon the patients' dinner was served, consisting of turkey (another gift from a kind donor). The traditional plum pudding is necessarily absent from the dinner of the lying-in patient. The dinner was accompanied by a visit from some of the hospital officials, who exchanged cheery remarks with each poor woman, the baby being of course held up for admiration. At 1.30 o'clock the nurses and servants had dinner in their respective halls, when they partook of a hearty meal of genuine English roast beef and plum pudding.

LAMBETH INFIRMARY.—The wards were beautifully decorated with flowers, evergreens, and mottoes—the work of the nurses—and a very happy Christmas was spent by 580 patients. All seasonable fare was most generously and abundantly granted by the Guardians, and much enjoyed by the patients. Christmas cards were distributed by Miss Griffiths, the Matron, and Miss Tomkins the Assistant Matron, and each child received sixpence, the gift of the Editor of *Truth*, who had also sent his usual kind gift of toys. During the afternoon and evening the patients were entertained with singing, &c., by the nurses, who, as a patient afterwards remarked, "Sang as sweetly as if there was no fog, and as if they was'nt a bit tired."

AT the ABBEY POORHOUSE, PAISLEY, there was a grand Christmas dinner to the 80 patients, and in the evening a soirée, over which the Chairman of the House Committee presided. Great praise is due to the nursing staff for their kindness in decorating the hall and otherwise entertaining the patients, also to the Matron. During the evening an excellent programme was gone through, consisting of Christmas anthems and sacred songs.

THE ESSEX AND COLCHESTER HOSPITAL was as usual very tastefully decorated for Christmas. Festoons, evergreens, and Chinese lanterns were carefully arranged in the several wards and passages in such a manner as to produce a very striking effect. Fairy lamps were also displayed to advantage on tables, and the numerous devises, composed chiefly of evergreens worked on a scarlet ground, reflected great credit on the nurses and others who assisted in carrying out the work of decoration. The day was spent as usual, a service being held in the afternoon. After tea at five o'clock, an excellent entertainment, organised by Mr. C. E. White, was given in one of the wards, and was much enjoyed. The Matron, House Surgeon, and Dr. Wallace, were lustily cheered by the audience. The National Anthem, in which the entire company joined, closed a pleasant evening.

THE Christmas festivities at the CITY ASYLUM, BIRMINGHAM began on Christmas Eve, when a ball took place in the dining hall. On Christmas Day the patients attended chapel, and afterwards had a dinner of roast beef, plum pudding, and dessert of various kinds. The children had a Christmas tree provided for them, hung with sweets and toys; the afternoon was spent in playing games and music. On Monday evening, dancing and music; Wednesday, a dramatic performance; and on New Year's Day, another dinner of roast beef and plum pudding.

The Matron of the COTTAGE HOSPITAL, LYME REGIS, writes: You will be glad to know that we had a very happy Christmas here; we had some little amusements before Christmas, and intend to have some more. Christmas Day was a really jolly day, lots of presents had been sent by kind friends, clothing for patients, flowers, fruit, evergreens, cakes, game, vegetables, &c. At breakfast time each patient had given to them a parcel containing an article of clothing, a book, and cards; during the afternoon their friends came, had tea, and remainded the evening; all appeared to enjoy themselves thoroughly. I must tell you of two new things we had for their amusement, a lot of parcels, of every size and shape; they were put in a place where everyone could see them in order that they might decide from the look of the outside which parcel they would like to have. After tea they were distributed, or rather, each one (patients and friends) took the one they fancied, the size of it had nothing to do with the article inside, because they were mostly padded with waste paper; one large parcel (a box), chosen by a man, when opened, displayed a wooden doll, which caused great fun; some of them had nice things in, and some had only toys.

The annual Christmas entertainment to the patients and nurses of the OLDHAM INFIRMARY took place on New Year's eve, and was of an unusually bright and successful character. The various wards, which had been most tastefully decorated with flags and evergreens, presented a most inviting appearance, and excited great admiration among the visitors. The proceedings commenced at 6 p.m. with a huge Christmas tree, heavily laden with toys and useful presents of every description; these were distributed among the patients, after which the guests, who had come in large numbers, and as many of the patients as could safely be moved, repaired to the out-patients' room, where a stage had been erected. A series of interesting and artistic tableaux, interspersed with vocal and instrumental music, were there given by some of the residents in the town. A very amusing exhibition of Mrs. Jarley's Waxworks terminated the evening's entertainment. The Matron, Miss Thompson, with her usual tact and amiability, was indefatigable in her efforts to make everyone feel at home, and to her the greatest credit is due for one of the most pleasant and agreeable gatherings that have ever taken place at this infirmary. We must not omit to mention Dr. McCraith, the House Surgeon, to whose great exertions the success of the entertainment was also in a great measure due.

### Everybody's Opinion.

### NOTICE OF APPOINTMENTS.

"Matron" writes: Having seen in The Hospital the letter from "Brisbane" respecting the uncourteous behaviour of the Secretary (or possibly Committee) of the Rotunda Hospital, Dublin, I, too, can testify to the same treatment. I was daily expecting either to be summoned before the Election Committee, or to receive notice of the post being filled, but have not to this day heard from the Secretary, or had the copies of my testimonials returned. This kept me in such an unsettled state that had I heard of any other appointment at the same time I should not have applied for it. I should like to know how many candidates were summoned for the election, or how, or when the appointment was made, and consider that, in justice to the applicants, whom I doubt not were many, a full explanation should be given in The Hospital, in which paper the advertisement for a Lady Superintendant appeared, and to which, at least, a notice of her appointment might have been sent. For women now-a-days who have to earn their living the struggle is hard enough, without being hindered by the rude and unbusiness-like ways of those who ought to know better. I am glad to say that this is the first time I have experienced such treatment from a hospital secretary or committee, and hope, for the honour of such institutions, which I do not like to hear reproached, that it is not the method of business of many of them.

### Death in Our Ranks.

It is with great regret we have to announce the death of Miss Lucy Osburn which occurred at Harrogate at the residence of her sister, on December 22nd. Miss Osburn was trained at St. Thomas's Hospital. She was appointed the first Matron of the Sydney Hospital, New South Wales. She may justly be called the pioneer of trained nursing in Australia. In spite of great difficulties she raised the standard of nursing to what it ought to be, and after 17 years' devoted work she returned to England, leaving the nursing in Sydney, and in the many institutions and hospitals she was the means of supplying with nurses, well organised, and in a thoroughly satisfactory condition. Since her return to England she has devoted herself to district nursing among the sick poor until the spring of the present year, when her health broke down. Miss Osburn was a most earnest-hearted, unselfish worker, and her work was done in a most unostentatious manner. Only her most intimate friends knew how entirely her life was spent for others. Several beautiful wreaths and crosses are on her grave in the little country churchyard where she is laid to rest, and amongst them we noticed one sent "In affectionate remembrance from her old Nurses of Sydney Hospital," some of whom are now in this country.

### Presentations.

Cottage Hospital, Congleton.—Miss Wagnell, the Matron, was presented by patients and friends with an illuminated address, a timepiece, and gold brooch on the 24th ult., being the occasion of the annual Christmas festivities at this hospital.

The North Cambs. Hospital.—Miss M. E. Lucas, Matron, was on Christmas Day presented with a chased set of "Apostle spoons" by the honorary surgeons connected with the hospital, as a mark of respect and esteem."

On Christmas Eve Miss Emery, Matron of the Nurses' Home, Newcastle-on-Tyne, was presented with an antique silver tea service by the nursing staff, with their love and good wishes.

At the Nurses' Co-operation on Christmas Day the nurses presented Miss K. Philippa Hicks with a diamond and sapphire ring, and Miss Leigh with an art necklace. Two of the nurses also took flowers to Dr. Hadden and Mr. Pitts, the hon. physician and hon. surgeon to the Co-operation.

On Christmas Day the nurses of the Royal Derby and Derbyshire Training Institution presented the Lady Superintendent, Miss E. A. Woodhead, with a silver Queen Anne teapot and Crown Derby stand, together with a card in leather case containing the names of the donors and the following inscription: "The accompanying Silver Teapot and Stand are presented to Miss Woodhead by the undersigned as a small token of their affection and esteem. Christmas, 1891."

As a pleasing testimony of the esteem in which Miss Hardy, Lady Superintendent of the West London Hospital, is held by her nursing staff, she was presented on Christmas Day by the Sisters and nurses of the hospital with a large handsome photographic album, and an artistic lamp shade, carried out in tones of pale yellow. The names of the donors were inscribed on the fly-leaf of a pretty illuminated copy of "Longfellow's Psalm of Life," which accompanied the other gifts.

### Where to Go.

The first annual meeting of the Nurses' Co-operation will be held at 4 p.m., on January 19th, at the rooms of the Medical-Chirurgical Society, 20, Hanover Square, W., Dr. Broadbent in the chair.

A course of St. John's nursing lectures will commence at 17, Old Cavendish Street, on January 26th, at 8.30 p.m. Fee 7s. 6d.

The annual social gathering of the Nurses' Union will be held on Thursday, January 14, at Morley Hall, 316, Regent Street, from 3–9 p.m. Any trained nurses who would care to attend, are invited to do so.

The General Meeting of the members of the Midwives' Institute and Trained Nurses' Club will be held on Friday evening, January 29th, at six o'clock, to be followed by a social tea at seven o'clock given by a member of the Committee, and to which all members are invited. The Secretary requests those members who intend to be present to kindly send her a post-card to that effect not later than the 25th inst.

### Amusements and Relaxation.

| Names. | Dec. 31st. | Totals. |
|---|---|---|
| Lightowlers | 35 | 573 |
| Bonne | 35 | 574 |
| Morico | 36 | 643 |
| Robes | — | 143 |
| Dulcamara | 37 | 588 |
| Psyche | — | 7 |
| Agamemnon | 33 | 610 |
| Nurse J. S. | 37 | 550 |
| Jenny Wren | 25 | 485 |
| Darlington | 35 | 569 |
| Nurse G. P. | — | 99 |
| Hetty | 34 | 485 |
| Janet | — | — |
| Jackanapes | — | 367 |
| Ex-Nurse | — | 99 |

**Results of Fourth Quarterly Word Competition.**

*First Prize*, 15s., is awarded to Morico (Mdile. M. M. Didier d'Ambléon, The Dispensary, Jersey).

## Figures and Tables

**Fig. 1. f1:**
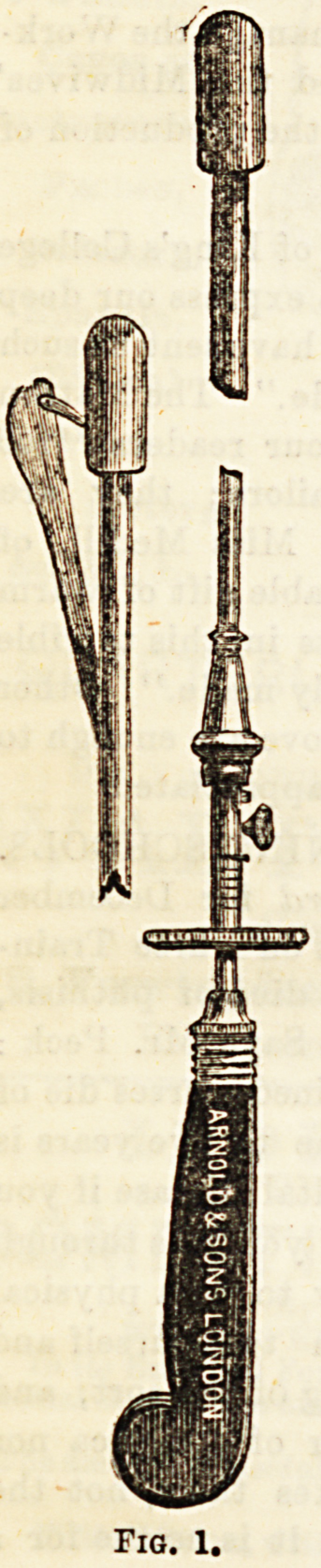


**Fig. 2. f2:**
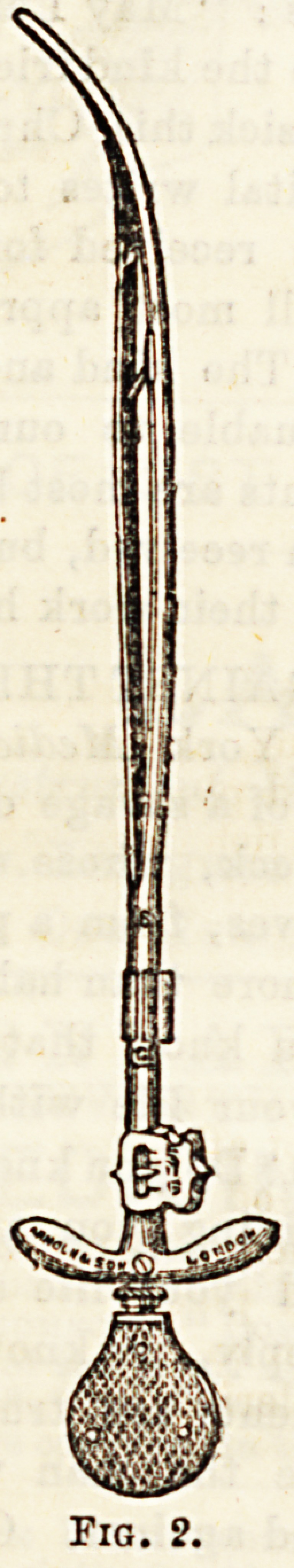


**Fig. 3. f3:**
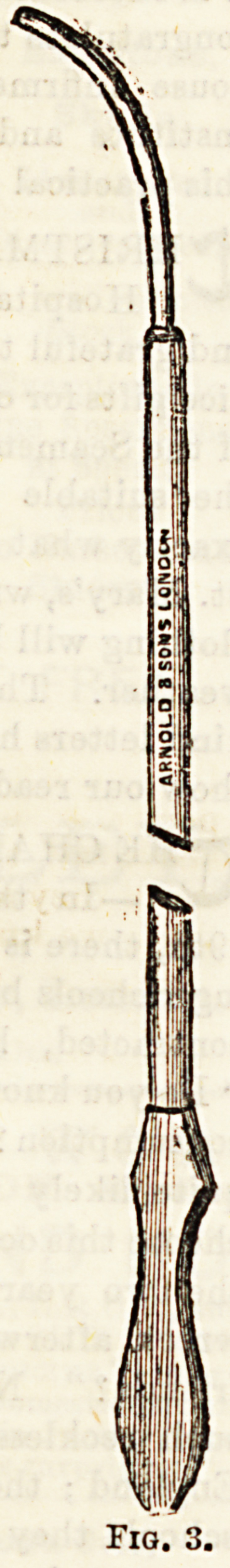


**Fig. 4. f4:**
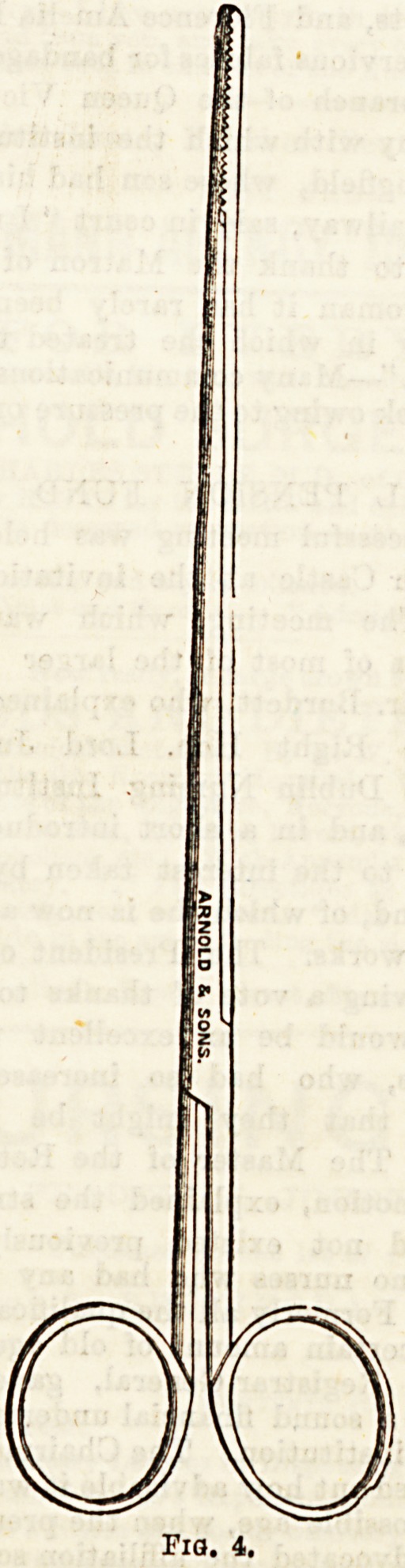


**Fig. 5. f5:**
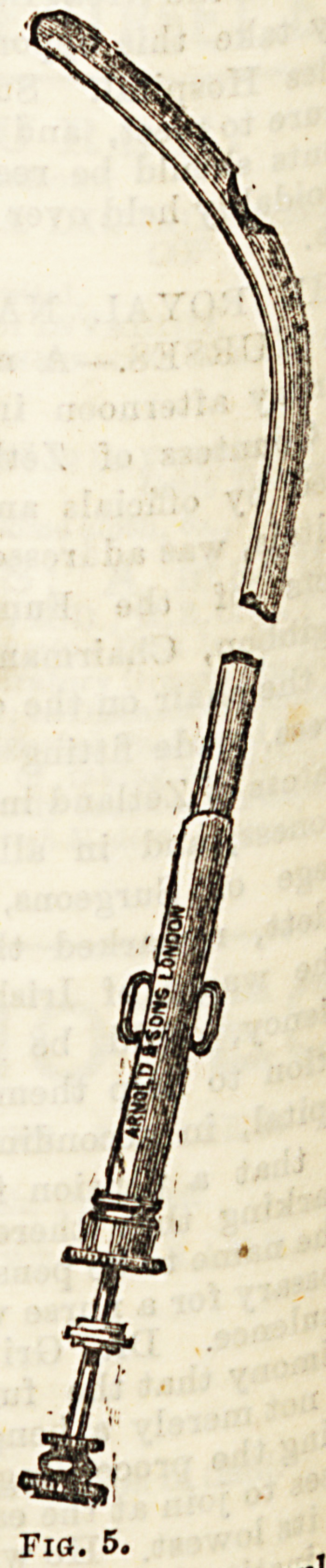


**Fig. 6. f6:**
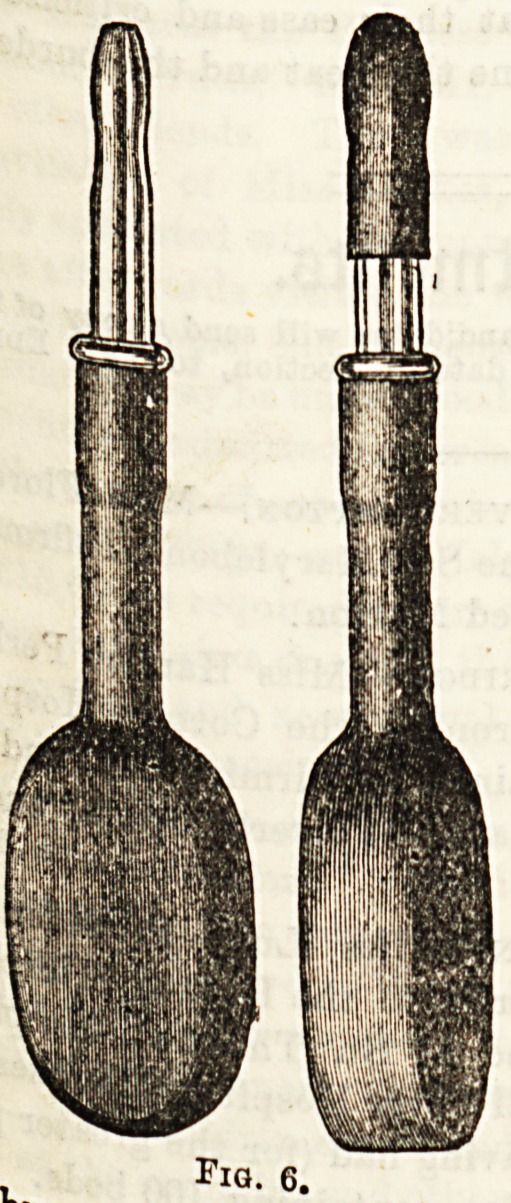


**Fig. 7. f7:**
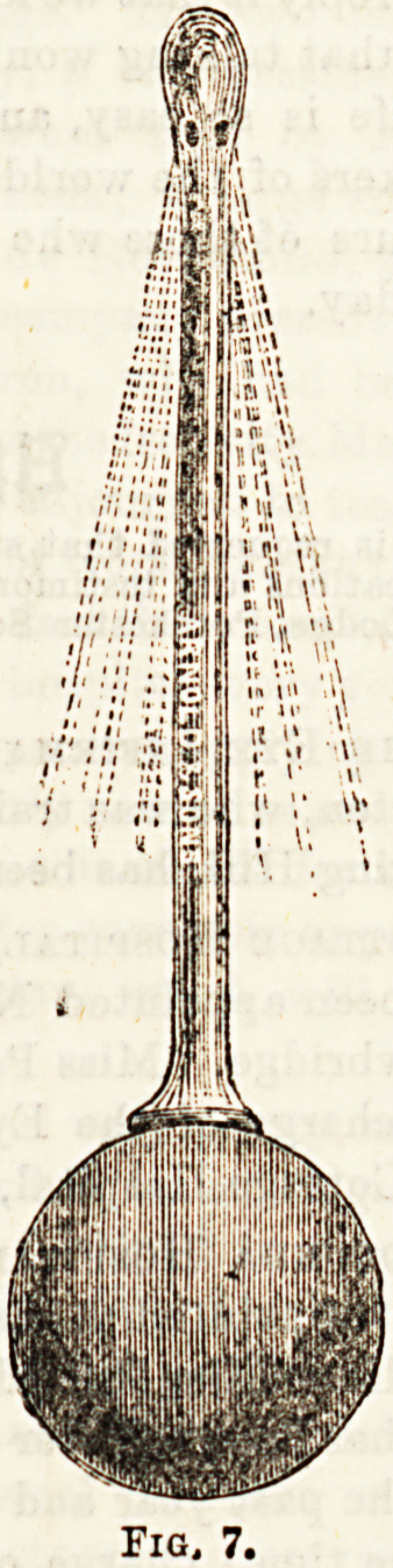


**Figure f8:**